# Mechanism Underlying the Shading-Induced Chlorophyll Accumulation in Tea Leaves

**DOI:** 10.3389/fpls.2021.779819

**Published:** 2021-12-02

**Authors:** Jiaming Chen, Shuhua Wu, Fang Dong, Jianlong Li, Lanting Zeng, Jinchi Tang, Dachuan Gu

**Affiliations:** ^1^Guangdong Provincial Key Laboratory of Applied Botany & Key Laboratory of South China Agricultural Plant Molecular Analysis and Genetic Improvement, South China Botanical Garden, Chinese Academy of Sciences, Guangzhou, China; ^2^College of Life Sciences, University of Chinese Academy of Sciences, Beijing, China; ^3^Guangdong Food and Drug Vocational College, Guangzhou, China; ^4^Laboratory of Tea Plant Resources Innovation and Utilization, Tea Research Institute, Guangdong Academy of Agricultural Sciences & Guangdong Provincial Key, Guangzhou, China

**Keywords:** shading, light intensity, chlorophyll accumulation, *Camellia sinensis*, transcription regulation

## Abstract

Besides aroma and taste, the color of dry tea leaves, tea infusion, and infused tea leaves is also an important index for tea quality. Shading can significantly increase the chlorophyll content of tea leaves, leading to enhanced tea leaf coloration. However, the underlying regulatory mechanism remains unclear. In this study, we revealed that the expressions of chlorophyll synthesis genes were significantly induced by shading, specially, the gene encoding protochlorophyllide oxidoreductase (*CsPOR*). Indoor control experiment showed that decreased light intensity could significantly induce the expression of *CsPOR*, and thus cause the increase of chlorophyll content. Subsequently, we explored the light signaling pathway transcription factors regulating chlorophyll synthesis, including CsPIFs and CsHY5. Through expression level and subcellular localization analysis, we found that CsPIF3-2, CsPIF7-1, and CsHY5 may be candidate transcriptional regulators. Transcriptional activation experiments proved that CsHY5 inhibits *CsPORL-2* transcription. In summary, we concluded that shading might promote the expression of *CsPORL-2* by inhibiting the expression of *CsHY5*, leading to high accumulation of chlorophyll in tea leaves. The results of this study provide insights into the mechanism regulating the improvements to tea plant quality caused by shading.

## Introduction

Tea plants, which are cultivated worldwide, are an important cash crop. Tea, which is made from their fresh shoots, is the most popular non-alcoholic beverage (Tang et al., 2019). The economic benefits of tea are closely related to its quality. In addition to the aroma and taste, color is a key trait used for measuring tea quality. The color of the dry tea leaves and the resulting liquid influences consumer preferences and purchasing decisions (Yu et al., 2019). The pigments that contribute to tea colors generally include chlorophyll, carotenoids, and anthocyanins, of which chlorophyll is the most important pigment for determining green tea quality (Suzuki and Shioi, 2003). As a traditional agronomic practice, shading is widely used in tea-producing countries, such as China and Japan. Considerable practical experience and scientific research have confirmed that shading can increase the overall quality of tea plants, both internally and externally (Chen et al., 2021). Moreover, shading enhances the taste and aroma of tea by affecting the synthesis of free amino acids, flavonoids, and aromatic compounds, while also affecting the appearance of tea by influencing the abundance of chlorophyll and other plant pigments as well as structural components (e.g., lignin; Ku et al., 2010; Liu et al., 2017; Zeng et al., 2020; Chen et al., 2021; Teng et al., 2021; Zhao et al., 2021). During tea plant cultivation, providing plants with sufficient shade can significantly improve tea color quality. To produce Japanese matcha, tea plants are often shaded by 60–98% for 10–30 days (Yamashita et al., 2020). The chlorophyll content of tea leaves increases significantly after shading treatment. Compared with the dry leaves of tea plants grown without shading, the dry tea leaves of plants grown in the shade are greener, which increases their economic value (Sano et al., 2018). To date, considerable research has been conducted on the phenomenon that shading improves tea quality. However, the research has focused primarily on the mechanism underlying the changes in the chemical components that affect sensory quality, including the regulation of flavonoid and free amino acid contents (Lee et al., 2013; Chen et al., 2017; Liu et al., 2018). There have been relatively few studies on the molecular basis of shading-induced chlorophyll accumulation.

Previous studies revealed that significant increases in the chlorophyll content of fresh leaves due to shading are unaffected by leaf age, shading season, and number of shading treatments but are related to the duration and degree of shading (Lee et al., 2013; Sano et al., 2018; Yamashita et al., 2020). Using an electron microscope, Liu observed that shading induces the development of tea leaf plastids, leading to an increase in the number of chloroplasts and in thylakoid compactness (Liu et al., 2020). Chlorophyll is the main pigment for photosynthesis in plants, so the synthesis of chlorophyll is a basic metabolic process (Garrone et al., 2015). In all oxygen-producing photosynthetic organisms, the light-dependent protochlorophyllide oxidoreductase (POR) enzyme is the key regulatory enzyme in the chlorophyll synthesis pathway (Lebedev and Timko, 1998; Masuda and Takamiya, 2004; Heyes and Hunter, 2005; Reinbothe et al., 2010; Scrutton et al., 2012). In one of the final steps, POR catalyzes the light-driven reduction of protochlorophyllide (PChlide) to chlorophyllide, from which chlorophyll is eventually derived (Garrone et al., 2015). For example, light-grown *Arabidopsis porB*/*porC* double mutants develop a seedling-lethal and chlorophyll-deficient phenotype (Frick et al., 2003). Additionally, rice *fgl* mutant (*OsporB* mutant) etiolated seedlings contained smaller prolamellar bodies in etioplasts, and lower levels of total and photoactive Pchlide (Sakuraba et al., 2013). However, how shading affects the chlorophyll synthesis pathway and how to regulate *POR* in tea leaves is not yet known.

Two types of shading are used during the cultivation of tea plants, namely, ecological shading and cover shading. Ecological shading involves the planting of trees in tea gardens or intercropping with economically valuable forest trees or tall and suitable tree species to provide shade. Cover shade is mainly provided by plastic greenhouse covers and sunshade net covers made from diverse materials, including straw, wheat straw, and artificial materials (Chen et al., 2021). Although there are various ways of shading plants, they generally alter environmental factors similarly. For example, they usually decrease the temperature and light intensity, change the light quality, and increase the environmental humidity (Fiorucci and Fankhauser, 2017). Among them, the change of light environment caused by shading is an important environmental factor that affects the synthesis of plant chlorophyll. In plants, light is required for photosynthesis and normal growth and development. Shading decreases the light intensity and the red (R) light: far-red (FR) light ratio, which affects plant growth and development (Fiorucci and Fankhauser, 2017). During long-term evolution, plants developed a variety of photoreceptors that perceive changes in the external light environment, including phytochromes that perceive R and FR light, cryptochromes and phototropins that perceive blue and ultraviolet A light, and UVR8, which perceives UVB light (Jiao et al., 2007; Rizzini et al., 2011). After the light signal is sensed by these photoreceptors in the plant, the downstream transcription cascade involving transcription factors is then initiated to regulate the growth and development of the plant (Jiao et al., 2007). Among these transcription factors, phytochrome-interacting factors (PIFs) and HY5 are critical for multiple pathways that integrate light signals and phytohormone signaling pathways, with key roles related to plant growth and development (Paik et al., 2017). Earlier research proved that PIFs and HY5 have the opposite regulatory effects on chlorophyll biosynthesis (Chen et al., 2004). More specifically, PIFs accumulate in seedlings grown in darkness and negatively regulate the expression of chlorophyll biosynthesis genes to inhibit the greening of seedlings (Moon et al., 2008). In contrast, HY5 regulates nuclear gene transcription and promotes seedling photomorphogenesis (Kindgren et al., 2012). Although chlorophyll synthesis has been thoroughly analyzed in model plants, the molecular regulation of chlorophyll biosynthesis in woody plants is poorly understood. For example, it is unclear how shading induces the accumulation of chlorophyll in tea plants. In recent years, the development of omics technology has further deepened our understanding of plant growth and developmental events. By applying transcriptomics technology, Liu determined that shading alters *PIF* and *HY5* expression patterns in tea plants (Liu et al., 2018). Accordingly, PIFs and HY5 may have regulatory functions, but the precise roles of these transcription factors in developing tea plants will need to be elucidated.

Because of a lack of an established genetic transformation system applicable for tea plants, there has been limited research on the molecular mechanism underlying the shade-induced regulation of chlorophyll synthesis. Therefore, the objective of this study was to clarify the regulatory effects of shading on the chlorophyll synthesis of tea plants. The effects of shading on tea plants were investigated under tea garden conditions and under simulating shading indoors conditions by decreasing the light intensity. We explored whether the decreased light intensity caused by shading is the main cause of the accumulation of chlorophyll in tea leaves. Additionally, the expression of genes encoding key rate-limiting enzymes of the chlorophyll synthesis pathway and the transcription factors involved in the light signaling pathway were analyzed. Finally, the regulatory mechanism was analyzed *via* subcellular localization and transcriptional activation experiments. The results of this study provide insights into the regulation of tea secondary metabolism and may be useful for the breeding and selection of high-chlorophyll tea varieties.

## Materials and Methods

### Sample Processing

Tea plants “Jinxuan” (containing almost 400 branches), which were 15 years old and grew consistently in tea garden, were selected to perform 2 weeks shading treatment. The shading treatment was carried on the Tea Research Institute, Guangdong Academy of Agricultural Sciences (Yingde, Guangdong, China) and started on August 2020 and March 2021. The tea plants in the treatment group (T) were covered by black shading nets for 2 weeks, the shading rate was 90%, and the light intensity was about 130 μmol m^−2^ s^−1^. The control group (CK) was not shaded, and the light intensity was about 1,300 μmol m^−2^ s^−1^. The branches with one bud and three leaves were collected 1st day, 7th day, and 14th day after treatment. Every five branches were randomly collected from the 400 branches combined into a group as one replicate. Three replicates were randomly collected from each treatment. The collected branches were immediately frozen in liquid nitrogen and stored at −80°C for subsequent RNA extraction and chlorophyll analysis.

The annual “Jinxuan” cutting seedlings with consistent growth were selected for the indoor control experiment, which started in May 2021. The tea seedlings were cultured in a light incubator at 25°C and the photoperiod was 16 h/8 h. The light source is the incubator’s own light source. The light intensity in the control group (CK) was higher than that in the tea garden treatment group (T), which was 252 μmol m^−2^ s^−1^. The light intensity in the experimental group was lower than that in the tea garden treatment group (T), which was 16.8 μmol m^−2^ s^−1^. After the treatment for 21 days, the branches with one bud and three leaves were collected. Every five branches were combined into a group as one replicate. Three replicates were randomly collected from each treatment. The collected branches were immediately frozen in liquid nitrogen and stored at −80°C for subsequent RNA extraction and chlorophyll analysis.

The indoor isolated branch control experiment was carried out in November 2020, and the branches used in the experiment were provided by the Tea Research Institute of Guangdong Academy of Agricultural Sciences. The branches pruned into one bud and three leaves and cultivated in a constant temperature and humidity environment. The cultivation temperature was 25°C and the photoperiod was 16 h/8 h. Light intensity was controlled by adjusting the number of white light tubes. Light quality was regulated by two external red or far-red LED tubes (Shenzhen FHT Electronics Technology Co., Ltd., Guangdong, China). The light intensity group experiment followed the principle of changing light intensity (light radiation) and unchanged light quality (the ration of R: FR). The light intensity for high, medium, and low-light intensity treatment was 252, 84, and 3.36 μmol m^−2^ s^−1^, respectively. The R: FR of all treatments was 1.5. The experiment of light quality group followed the principle of light quality (the ration of R: FR) change and light intensity (light radiation) unchanged. R: FR was 0.1 in high far-red light treatment and 7 in high red light treatment. The light intensity of all treatments was 252 μmol m^−2^ s^−1^. The light intensity of the control group was 252 μmol m^−2^ s^−1^, and the R: FR was 1.5. Taking into account that the isolated branches cannot survive for a long time, tea sample was collected after the treatment for 6 days. Every five branches were combined into a group as one replicate. Three replicates were randomly collected from each treatment. The collected branches were immediately placed in liquid nitrogen and stored at −80°C for subsequent RNA extraction and chlorophyll analysis.

### Chlorophyll Extraction and Content Determination

The extraction method of chlorophyll referred to the previous research method (Fu et al., 2018). The samples were ground into fine powder in liquid nitrogen, and chlorophyll of fresh sample powder (about 100 mg) was extracted with 5 ml of 80% acetone precooled at 4°C. After shaking and mixing, the powder was placed at 4°C for 12 h in a dark environment. After centrifugation at 10000 *g* for 10 min, the supernatant was taken to measure the absorbance (A_663_ and A_645_) and the chlorophyll content was determined.

The formula for calculating chlorophyll content was as follows (Arnon, 1949):


$$\mathrm{Chlorophyll}\;a=12.7\times A_{663}-2.69\times A_{645};$$

$$\mathrm{Chlorophyll}\;b=22.9\times A_{645}-4.68\times A_{663};$$

$$\mathrm{Total chlorophyll}=20.2\times A_{645}+8.02\times A_{663}.$$

### Analysis of Transcript Expression Levels of Related Genes

The fresh tea leaf sample was ground into a fine powder in liquid nitrogen, and the RNA was reverse transcribed into cDNA using PrimeScript^®^ RT Reagent Kit (Takara) according to the instructions. The target genes were detected using quantitative real-time polymerase chain reaction (qRT-PCR), and the primers required for qRT-PCR were designed by the Web site.^1^ The tested primers are shown in Supplementary Table S1. The qRT-PCR reaction system was as follows: 5 μl of iTaqTM Universal SYBR^®^ Green Supermix (Bio-Rad, Hercules, CA, United States), 2.5 μl of template cDNA (80 ng/μl), and 1 μm gene-specific primers (Supplementary Table S1) 2.5 μl. The qRT-PCR experiment was performed on Roche LightCycle 480 (Roche Applied Science, Mannheim, Germany). The specific procedure was as follows: 95°C for 30 s, 95°C for 5 s, and 60°C for 30 s, a total of 40cycles. *CsEF1-α* (GeneBank no. KA280301.1) was used as the internal reference gene. The melting point curve was tested to verify the specificity of PCR products. Three biological replicates for each sample were performed qRT-PCR analysis and each biological replicate had three technical repetitions. The 2^−△△CT^ method was used to calculate the relative expression, and the gene expression results were normalized.

### Cloning of CsPIF3-2/7–1 and CsHY5

*CsPIF3-2/7–1* and *CsHY5* gene sequence primers were designed (Supplementary Table S1) in reference to the NCBI database,^2^ and cDNA was used as a template. The PCR system was 20 μl. The PCR reaction program was as follows: denaturation at 98°C for 10 s, annealing at 56°C for 15 s, and extension at 72°C for 120 s, 30 cycles. The PCR product was detected and recovered, and the recovered product was ligated with the pHB-FLAG cloning vector and transformed into *E. coli* DH5α competent cells. Positive clones were selected for plasmid extraction and sequencing.

### Subcellular Location Assay

The CDS of *CsPIF3-2/7–1/7–2* and *CsHY5* without stop codon was cloned into pSAT6-EYFP vector. The *Arabidopsis* protoplast isolation method is as described before (Zhou et al., 2019). Leaf cells of *Arabidopsis* were enzymolyzed with cellulase and pectase at room temperature, filtered and washed with W5, and resuspended in ice bath for 30 min, and the constructed vector was transformed into protoplasts with PEG and incubated overnight with W5. The yellow fluorescence of CsPIF3-2/7–1/7-2-YFP and CsHY5-YFP was observed by confocal laser scanning microscopy (Zeiss LSM 510, Carl Zeiss, Jena, Germany). The primers used for subcellular vector construction were shown in Supplementary Table S1.

### Dual-Luciferase Reporter Assay

In order to detect the transcriptional activity of CsPIF3-2/7–1 and CsHY5 on *CsPORL-2*, we designed primers with reference to the promoter sequence of *CsPORL-2* (XM_028200153.1) in the NCBI database. The primers used for vector construction were shown in Supplementary Table S1. The promoter was cloned into pGreenII 0800-LUC dual-reporter vector (Hellens et al., 2005), and the constructed CsPIF3-2/7-1-FLAG and CsHY5-FLAG plasmids were used as effectors to infect tobacco leaves through *Agrobacterium* strain GV3101. After 2 d of co-transformation, the activities of LUC and REN luciferase were measured using a dual-luciferase reporting kit (Promega) and a microplate analyzer (Tecan Infinite F50, Tecan Aust-RIA GmbH). Due to the sequence of dual-reporter vector, when the effector has a transcription activity, the ratio of LUC to REN will change. The ratio of LUC to REN was to reflect the final transcription activity. Specifically, compared with the control group (co-transfection of the reporter with the empty vector), higher ratio indicates that effector has a role of transcription activation. Otherwise, lower ratio means the role of transcription inhibition. If there is no significant difference in LUC/REN, it indicates the effector may not transcription activity. At least three biological replicates were performed for each combination, which mean every combination were co-transformed into three tobaccos at least.

### Significance Analysis

The significant between two groups was determined based on paired and independent samples *t*-tests. One-way ANOVA combined with Duncan’s multiple range test was applied to figure out the differences among three groups. *p*≤0.05 was considered a significant difference, and *p*≤0.01 was a very significant difference. All statistical analyses were performed on an SPSS statistical package (Ver. 23.0).

## Results

### Shading Treatments-Induced Chlorophyll Accumulation and the Related Gene Expression in Tea Leaves

**Figure 1:**
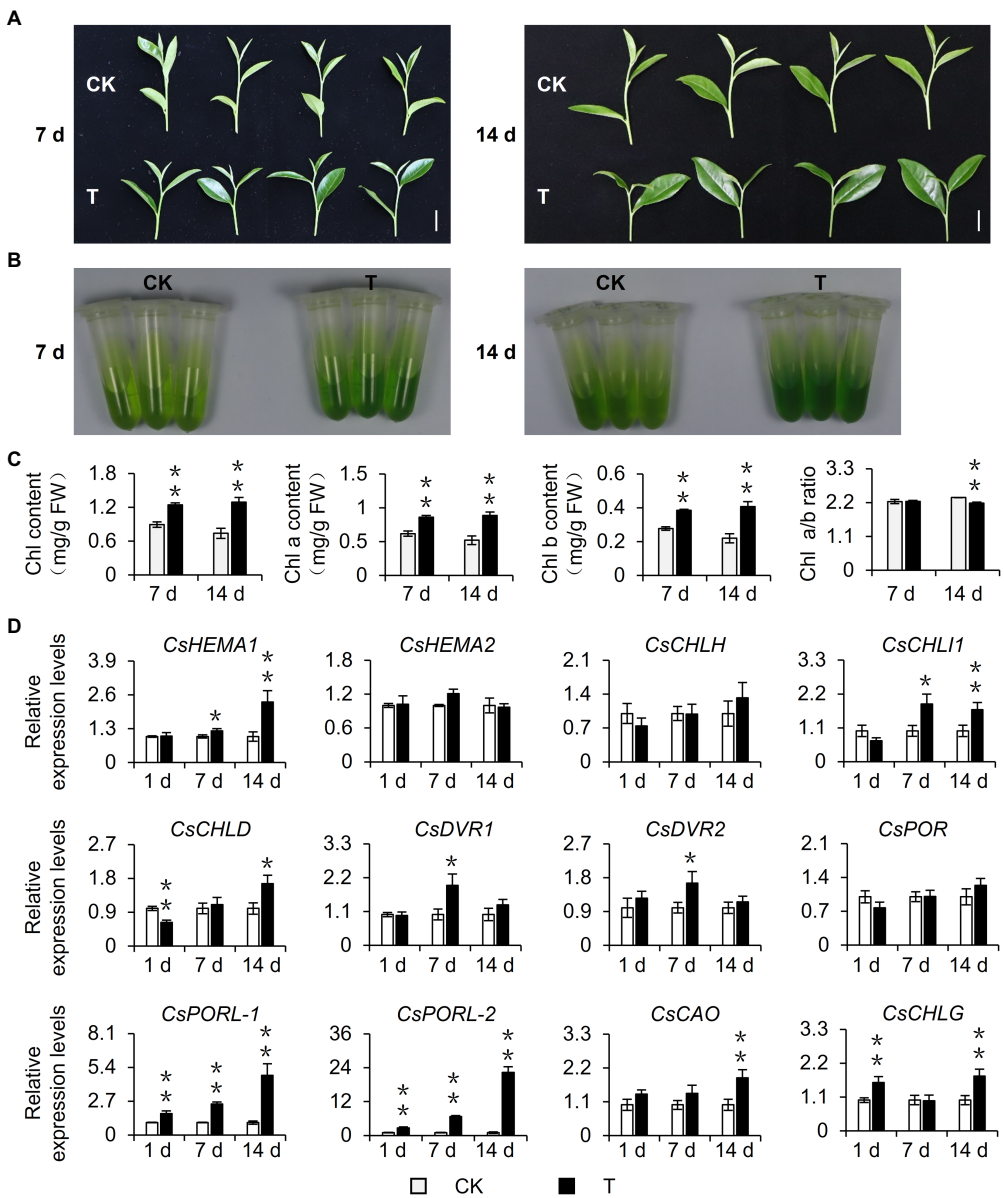
Effect of shading treatment on the chlorophyll content of tea leaves and the expression of related genes. **(A)** The phenotype of new shoots after 7 day and 14 day shading treatment. Bar=2 cm. **(B)** The comparison of leaf pigments after 7 day and 14 day shading treatment. **(C)** The change of chlorophyll content after shading treatment. Chl: total chlorophyll; Chl a: chlorophyll a; Chl b: chlorophyll b; and FW: fresh weight. **(D)** The changes in the gene expression of chlorophyll synthesis pathway after shading treatment. CK: no shading treatment group (about 1,300 μmol m^−2^ s^−1^); T: 90% shading treatment group (about 130 μmol m^−2^ s^−1^). *CsEF1-α* was used as an internal reference to normalized the changes. Data are expressed as mean±SD (*n*=3). ^*^p≤0.05; ^**^p≤0.01; and difference from CK treatment at the same time point.

To explore the effect of shading on the chlorophyll content of tea leaves, 15-year-old mature tea plants underwent a shading treatment in August 2020. Considering that current tea plant production methods often involve a 60–98% shading treatment and the fact that tea quality can increase substantially when plants are grown under 85% shade (Sano et al., 2018), the experimental group (T group) was grown under 90% shade. This shading treatment altered the tea plant phenotype. Specifically, the leaves were softer, larger (i.e., in terms of area), and were more intensely colored than the control leaves (Figure 1A). The leaf pigments were extracted with 80% acetone and the chlorophyll content was measured, which revealed the leaves of the shaded plants were greener than the control leaves (Figure 1B) because of a significant increase in the chlorophyll content. This shade-induced accumulation of chlorophyll was stable and increased as the number of shading treatment days increased. Additionally, this accumulation was reflected by the total chlorophyll content as well as the chlorophyll a and b contents. Interestingly, after plants were shaded for 14 days, the ratio of chlorophyll a: chlorophyll b decreased significantly, which may help plants capture light energy in a shaded environment (Chen et al., 2021; Figure 1C). Because the chlorophyll level is closely related to the expression of chlorophyll synthesis genes, we analyzed the shade-induced changes in the expression of genes associated with chlorophyll synthesis. The qRT-PCR analysis (Figure 1D) revealed that the shading treatment upregulated the expression of genes encoding key rate-limiting enzymes of chlorophyll synthesis, including *CsHEMA1*, *CsCHLH*, *CsCHLI1*, *CsCHLD*, *CsDVR1*, *CsDVR2*, *CsCAO*, *CsCHLG*, and especially *CsPOR* genes. Three *CsPOR* genes were identified in tea plants, namely, *CsPOR*, *CsPORL-1* (*CsPOR-like 1*), and *CsPORL-2* (*CsPOR-like 2*). The *CsPORL-2* transcription level in particular was significantly upregulated. Moreover, the results of leaves phenotype, chlorophyll contents, and *CsPORL-2* expression showed the same trends in the shade samples of March 2021 (Supplementary Figures S1A–C). Collectively, these findings indicate shading promotes chlorophyll synthesis in tea plants and regulates the chlorophyll content of new shoots, resulting in an increase in the intensity of the green coloration of the leaves.

### Decreased Light Intensity-Induced Chlorophyll Accumulation and the Related Gene Expression in Tea Leaves

**Figure 2:**
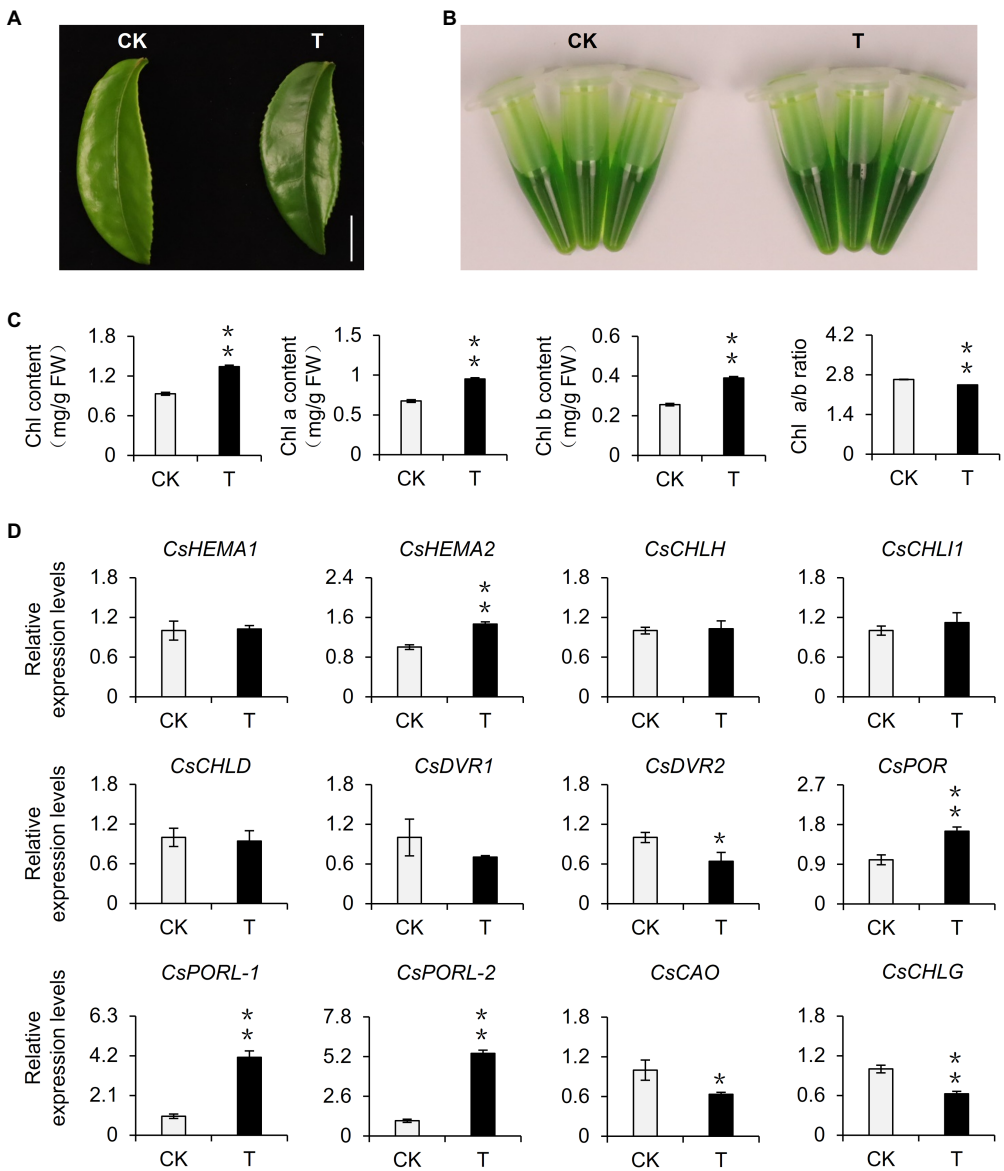
Effect of reduced light intensity on the chlorophyll content of tea leaves and the expression of related genes. **(A)** The phenotype of third leaf after different light intensity treatments. Bar=1 cm. **(B)** The comparison of leaf pigments after different light intensity treatments. **(C)** The change of chlorophyll content after different light intensity treatments. Chl: total chlorophyll; Chl a: chlorophyll a; Chl b: chlorophyll b; and FW: fresh weight. **(D)** The changes in the gene expression of chlorophyll synthesis pathway after different light intensity treatments. CK: 252 μmol m^−2^ s^−1^; T: 16.8 μmol m^−2^ s^−1^. *CsEF1-α* was used as an internal reference to normalized the changes. Data are expressed as mean±SD (*n*=3). Mean denoted by different sign indicates significant differences between the treatments in the same time (^*^mean *p*<0.05; ^**^mean *p*<0.01).

Shading can alter various environmental factors, including light intensity, light quality, temperature, and humidity, of which light intensity is closely related to the photosynthesis rate of plants and influences the accumulation of organic matter. In this study, the shading treatment caused the light intensity to decrease to only 10% of the light intensity of the control group. Therefore, we speculated that the decrease in light intensity caused by shading may be one of the environmental factors responsible for the phenotypic changes in tea plants. To eliminate the interference by multiple factors in the tea garden, we used annually cut seedlings to conduct indoor experiments, during which we simulated shading by decreasing the light intensity. After 21 days, we observed that the leaves were significantly greener than the control leaves (Figure 2A). This result was supported by analyses of the extracted leaf pigments (Figure 2B) and the chlorophyll content (Figure 2C). Additionally, the changes of the ratio of chlorophyll a: chlorophyll b were consistent with the results of the tea garden-shading experiments. Accordingly, the decrease in light intensity may be a key environmental factor inducing the accumulation of chlorophyll in tea leaves. We subsequently analyzed the differential expression of chlorophyll synthesis-related genes in the samples used for the indoor experiments. The decreased light intensity significantly upregulated the expression of *CsDVR2*, *CsPOR*, *CsPORL-1*, and *CsPORL-2*, which was consistent with the gene expression data for the tea garden-treated samples. Notably, the *CsPOR* transcription levels increased more stably and significantly in response to shading than the transcription levels of the other genes. In particular, *CsPORL-2* was very sensitive to shading and decreased light intensity, suggesting it may be a target gene that should be analyzed further.

**Figure 3:**
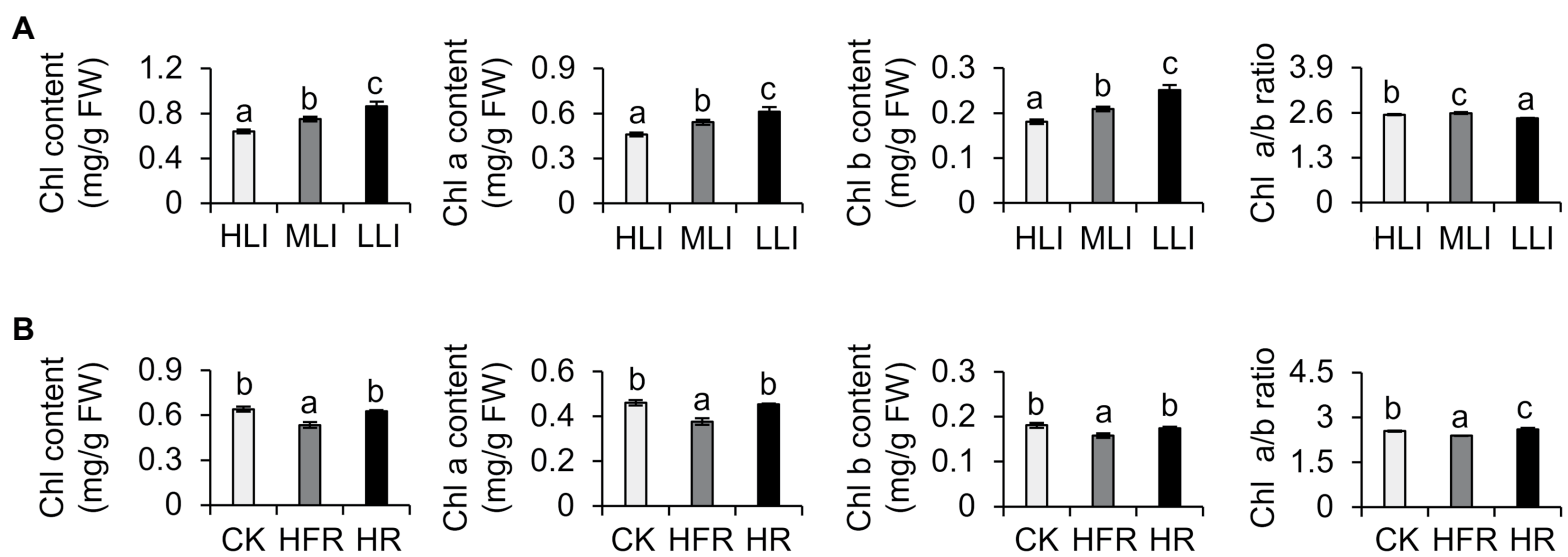
Effect of decreased light intensity and changes in light quality on the chlorophyll level of cut tea branches. **(A)** Changes in the chlorophyll levels of cut tea branches under decreased light intensity treatment. HLI: high light intensity treatment group (252 μmol m^−2^ s^−1^); MLI: medium light intensity treatment group (84 μmol m^−2^ s^−1^); LLI: low-light intensity treatment group (3.36 μmol m^−2^ s^−1^), and R: FR for all three groups were 1.5. **(B)** Changes in chlorophyll levels of cut tea branches after light quality changes. CK: R: FR was 1.5; HFR: high far-red group, R: FR was 0.1; HR: high red group, R: FR was 7, and the light intensity of the three groups was 252 μmol m^−2^ s^−1^. Chl: total chlorophyll; Chl a: chlorophyll a; Chl b: chlorophyll b; and FW: fresh weight. Data are expressed as mean±SD (*n*=3). Different letters represent significant differences between groups (*p*≤0.05) at the same time point.

Previous studies demonstrated that shading modulates light intensity but affects light quality (e.g., R: FR; Fiorucci and Fankhauser, 2017). To verify our results, we also explored the effects of changes in light intensity and light quality on the chlorophyll synthesis in detached branches. Our analysis indicated that decreasing light intensity can induce the accumulation of chlorophyll in new shoots (Figure 3A). Moreover, the change in light quality also affected the chlorophyll content. For example, the chlorophyll level decreased significantly under high-FR light conditions (R: FR=0.1; Figure 3B). Thus, the decrease in light intensity caused by shading was the main environmental factor that induced the accumulation of chlorophyll in tea leaves, but the change in light quality also affected the chlorophyll content to some extent.

### Shading and Reduced Light Intensity Regulated the Expression of Genes Related to the Light Signaling Pathway in Tea Plants

**Figure 4:**
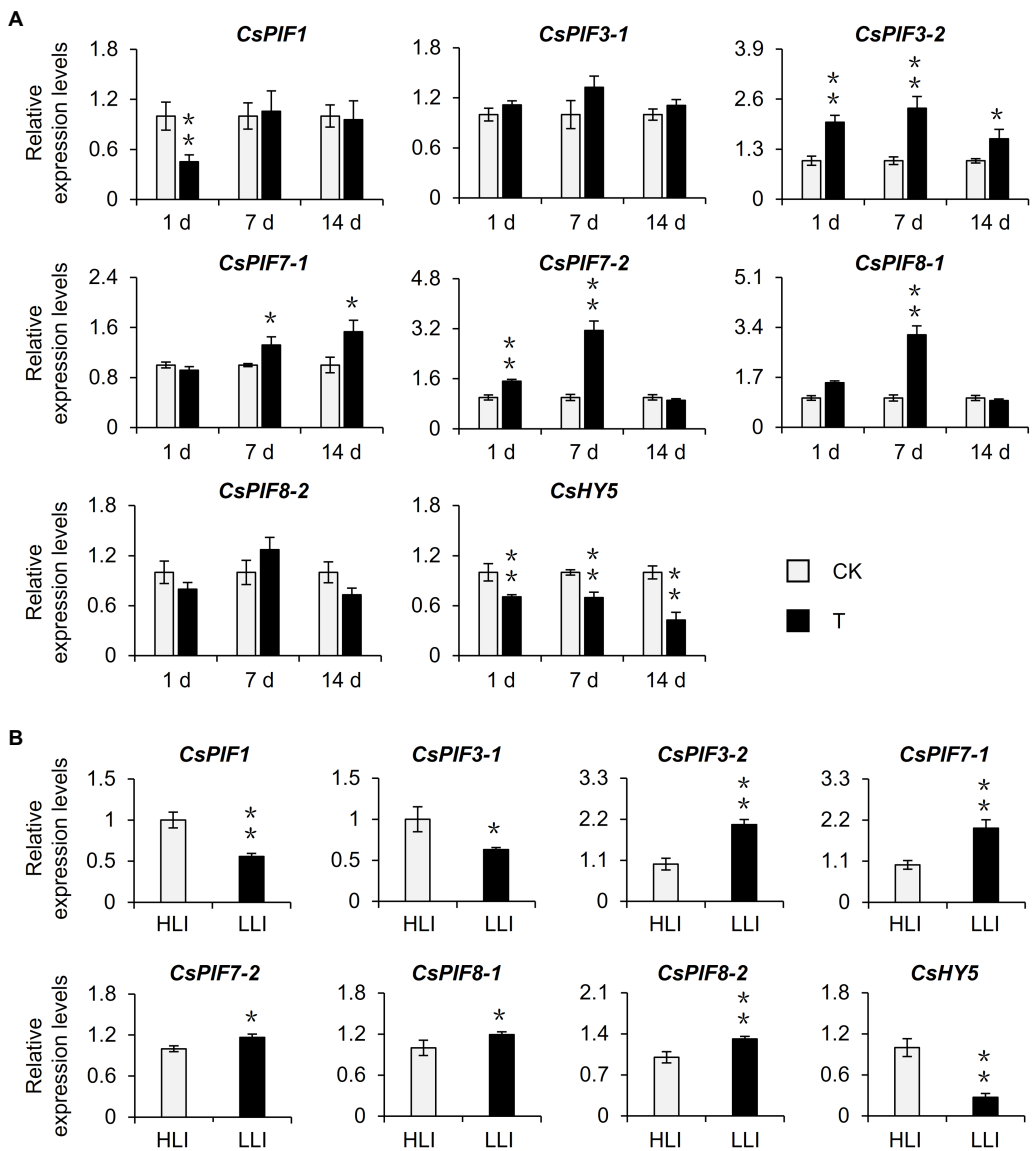
Effects of shading and reduced light intensity on the expression of genes related to the light signal pathway in tea plants. **(A)** Changes in the expression of genes related to light signal in tea plants under shading treatment. CK: no shading treatment group (about 1,300 μmol m^−2^ s^−1^); T: 90% shading treatment group (about 130 μmol m^−2^ s^−1^). **(B)** Changes in the expression of light signal-related genes in tea plants under reduced light intensity. HLI: high-light intensity treatment group (252 μmol m^−2^ s^−1^); LLI: low-light intensity treatment group (16.8 μmol m^−2^ s^−1^), and R: FR for two groups were 1.5. *CsEF1-α* was used as an internal reference to normalized the changes. Data are expressed as mean±SD (*n*=3). ^*^p≤0.05; ^**^p≤0.01; and difference from CK treatment at the same time point.

Previous studies identified PIFs and HY5 as important regulators of the light signaling pathway and confirmed their importance to chlorophyll synthesis (Chen et al., 2004; Paik et al., 2017). To further screen the potential target transcription factors affected by shading, we examined the transcription levels of *CsPIFs* and *CsHY5* homologs in the shaded samples in the tea garden. The shading treatment significantly induced the expression of *CsPIF3-2*, *CsPIF7-1*, *CsPIF7-2*, and *CsPIF8-1* in tea plants, whereas it had the opposite effect on *CsHY5* expression (Figure 4A, Supplementary Figure S1D). A similar gene expression analysis of the indoor cut seedling samples confirmed that *CsPIF3-2*, *CsPIF7-1*, *CsPIF7-2*, and *CsHY5* expression levels were affected by the changes in light intensity, with expression trends that were consistent with those observed for the shaded samples in the tea garden (Figure 4B). Moreover, the transcription factor gene expression levels were changed as the light intensity decreased, implying that shading may influence the expression of *CsPIF3-2*, *CsPIF7-1*, *CsPIF7-2*, and *CsHY5* in tea plants because of the associated decrease in light intensity, thereby regulating the transcription of the downstream chlorophyll synthesis genes.

### Subcellular Localization of CsPIF3-2/7–1/7–2 and CsHY5

**Figure 5:**
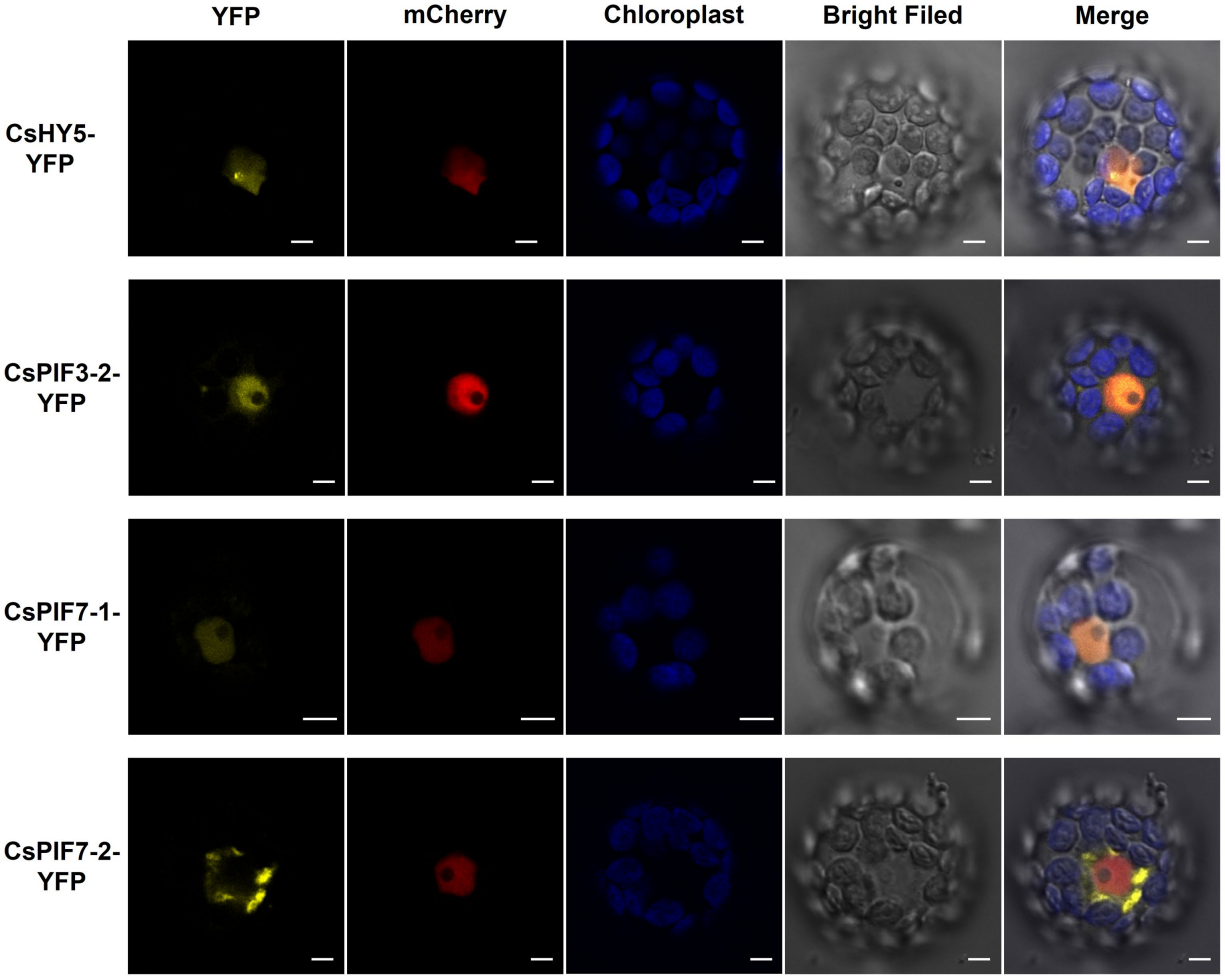
Subcellular localization of CsPIF3/7 and CsHY5. The YFP fusion protein of CsPIF3/7 and CsHY5 was transiently expressed in *Arabidopsis* protoplasts. Yellow fluorescence represented YFP fusion protein of CsPIF3/7 and CsHY5. Red fluorescence represented mCherry protein marker for cell nucleus. Blue fluorescence was the chlorophyll autofluorescence. CsHY5-YFP, CsPIF3-2-YFP, and CsPIF7-1-YFP fusion protein were visible in nucleoplasm structures in the *Arabidopsis* leaf cell. CsPIF7-2-YFP fusion protein may be not in nucleoplasm structures. Bar=5 μm.

The tea garden-shading experiments and the indoor experiments involving decreased light intensity revealed the transcription factor genes *CsPIF3-2*, *CsPIF7-1*, *CsPIF7-2*, and *CsHY5* may encode key regulators of chlorophyll accumulation in shaded plants. These transcription factors may modulate chlorophyll synthesis by regulating the expression of genes related to chlorophyll synthesis. To further characterize the functions of these transcription factors, we first analyzed their subcellular localization using *Arabidopsis thaliana* protoplasts. The results indicated that CsPIF3-2, CsPIF7-1, and CsHY5 were located in the nucleus (Figure 5), suggesting they may contribute to the regulation of *CsPOR* expression. However, CsPIF7-2 was not detected in the nucleus, indicating it may not have a direct regulatory role related to *CsPOR* transcription (Figure 5).

### CsPIF7-1 and CsHY5 Regulated the Transcription of Chlorophyll Synthesis Genes

**Figure 6:**
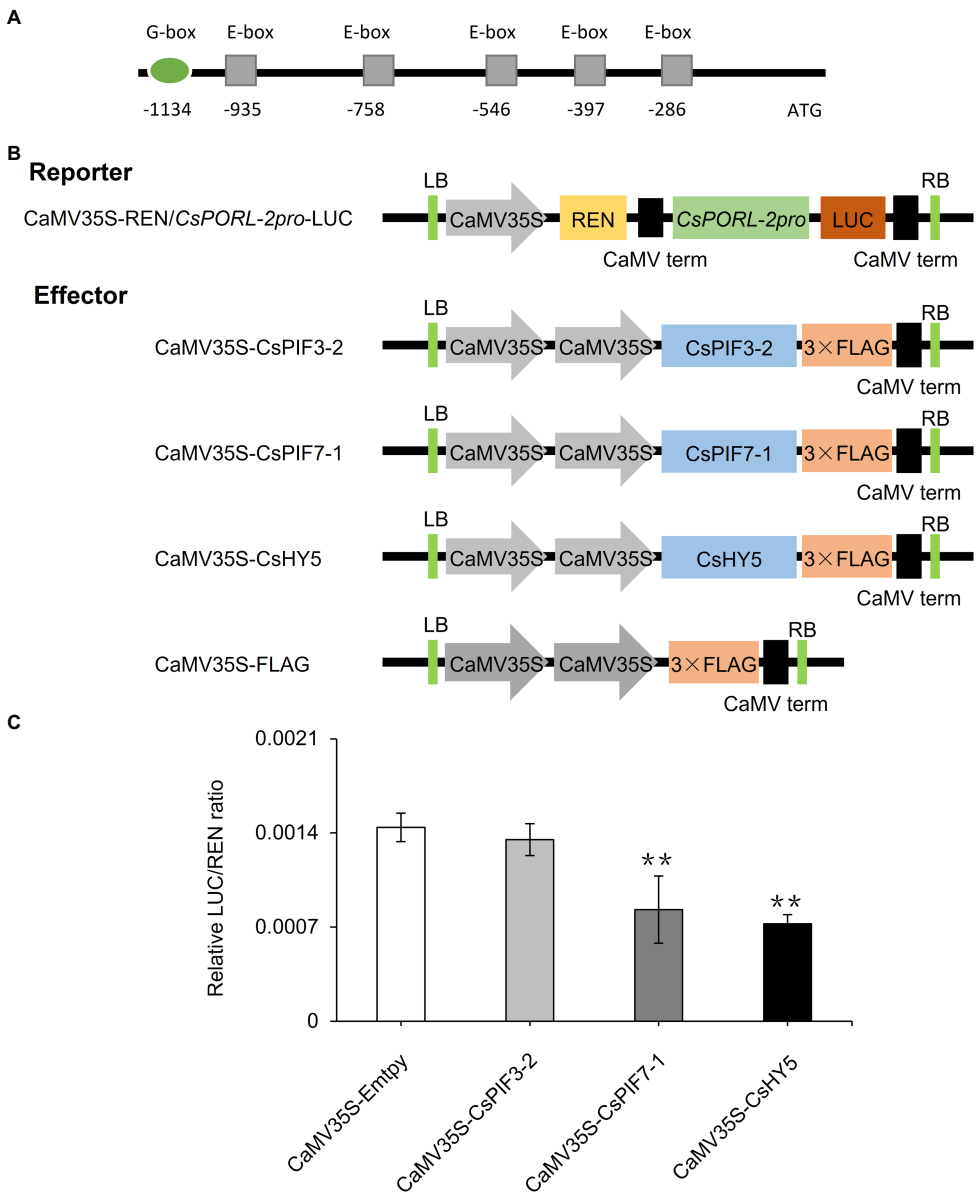
Involvement of CsPIF3-2/7–1 and CsHY5 in regulating the expression of genes related to chlorophyll synthesis. **(A)** *CsPORL-2* promoter element analysis. **(B)** Schematic representation of the double reporter and effector plasmids used in the dual-luciferase reporter assay. **(C)** CsPIF3-2, CsPIF7-1, and CsHY5 have transcriptional regulatory activity on *CsPORL-2* promoter. The regulation of *CsPORL-2* promoter by CsPIF3-2, CsPIF7-1, and CsHY5 was showed by the ratio of LUC to REN. Data are expressed as mean±SD (*n*=3). ^**^p≤0.01; and difference from empty-vector control group.

On the basis of analyses of metabolites, gene expression, and subcellular localization, we determined the effects of shading and decreased light intensity on the light signaling pathway transcription factor genes *CsPIF3-2*, *CsPIF7-1*, and *CsHY5* and the gene encoding the chlorophyll synthesis rate-limiting enzyme CsPORL-2. To further explore the regulatory effects of CsPIF3-2, CsPIF7-1, and CsHY5 on *CsPORL-2* expression, we first searched the tea plant reference genome and cloned the *CsPORL-2* promoter sequence. The detection of G-box and E-box promoter elements (Figure 6A) suggests that *CsPORL-2* expression may be directly regulated by CsPIF3-2, CsPIF7-1, and CsHY5. To clarify the regulatory mechanism, we examined the effects of CsPIF3-2, CsPIF7-1, and CsHY5 on the *CsPORL-2* promoter by conducting the dual-luciferase reporter assays. The dual-luciferase reporter plasmid harbor the *CsPORL-2* promoter fused to LUC, and the REN driven by the CaMV35S promoter as an internal control, while the plasmids expressing CsPIF3-2, CsPIF7-1, and CsHY5 as the effector (Figure 6B). As shown in Figure 6C, compared with the control, the LUC/REN ratio was remarkably decreased when CsPIF7-1 and CsHY5 were expressed, respectively. Moreover, no significant change in the LUC/REN ratio was found when CsPIF3-2 was expressed (Figure 6C). Collectively, these results revealed that CsPIF3-2 did not regulate *CsPORL-2* expression, both CsPIF7-1 and CsHY5 significantly inhibited *CsPORL-2* transcription.

## Discussion

Among the many quality-related traits improved by shading, pigmentation, including by chlorophyll, directly affects consumer preferences regarding tea leaves. In this study, we simulated shading in indoor experiments by decreasing the light intensity. The resulting chlorophyll accumulation was similar to that observed for plants shaded in the tea garden (Figure 2). Hence, decreasing light intensity may be one of the main factors associated with shading that promotes chlorophyll accumulation in tea leaves. Shading would cause changes in the micro-environment of tea tree growth, including changes in environmental factors, such as light intensity, light quality, temperature, and humidity (Fiorucci and Fankhauser, 2017). Studies on multiple species proved that temperature, light quality, and humidity affect plant chlorophyll contents (Bourque and Naylor, 1971; López-Figueroa and Niell, 1990; Huang et al., 2017). Investigations regarding celery indicated that heat and cold stresses can lead to decreased chlorophyll contents (Huang et al., 2017). An exposure to R light induces chlorophyll accumulation in seaweed (López-Figueroa and Niell, 1990). Although we did not prove that R light treatment of detached tea branches can induce chlorophyll accumulation, we determined that FR light treatment can significantly decrease chlorophyll levels (Figure 3). This suggests that light quality changes modulate tea plant chlorophyll synthesis, but changes in the light quality in a shaded environment may not be the main contributing factor. Additionally, highly humid environment reportedly promotes the accumulation of chlorophyll in Jack Bean (Bourque and Naylor, 1971), which is consistent with the effects of increasing environmental humidity due to the shading of tea plants. Therefore, the chlorophyll accumulation induced by the shading of tea plants may be the result of multiple factors associated with the improved micro-environment. Future investigations should focus on clarifying the effects of other environmental factors on chlorophyll accumulation in shaded tea plants. Moreover, the extent of shading influences the coloration of tea leaves. Tea plants generally grow normally in a shaded environment, with green leaves even at a 98% shading rate (Yang et al., 2012). In this study, we used 90% shading rate for the 2-week shading treatment of tea plants. The observed green coloration of the tea leaves (Figure 1) was consistent with the results of previous studies (Liu et al., 2020). However, if the degree of shading continues to increase, tea plants exposed to long-term shading (100% shading rate) will start to exhibit a yellowing phenotype, similar to model plants, such as *A. thaliana*. Chen et al. treated tea plants with 2weeks of shading (100% shading rate) and revealed the degradation of chlorophyll in tea leaves, which resulted in yellow leaves (Chen et al., 2017). Increasing the shading rate by as little as 2% can alter the tea leaf phenotype. Thus, the mechanism mediating the effects of different shading rates in the later periods should be explored.

Earlier research regarding the albino tea cultivar “Baijiguan” demonstrated that shading treatment causes leaves to turn green, increases the chlorophyll content, and facilitates the normal development of chloroplasts (Wu et al., 2016). Subsequent studies on the normal tea variety “Shuchazao” indicated that shading can increase the accumulation of chlorophyll, leading to the increased green coloration of leaves (Liu et al., 2018, 2020). In this study, we obtained similar phenotypic results during our outdoor shading treatment and indoor simulated shading treatment (i.e., decreased light intensity) of “Jinxuan” tea plants, which are widely cultivated in South China (Figures 1, 2). These findings prove that shading induces the accumulation of chlorophyll in tea leaves, including the leaves of albino varieties, implying tea plant responses to shading are conserved among varieties. This phenomenon may reflect an adaptive mechanism of tea plants, which are generally believed to be shade-tolerant species, to low-light environments. Omics-based analyses revealed that the expression of *CsPOR* genes, which encode protochlorophyllide oxidoreductases, is significantly induced in response to shading (Wu et al., 2016; Liu et al., 2020), whereas the expression of *CsHY5*, which is an important transcription factor gene in the upstream light signaling pathway, is inhibited (Wu et al., 2016; Liu et al., 2018, 2020). The results of the current study are in accordance with these earlier findings (Figures 1D, 4A). However, the relationship between *CsHY5* and *CsPOR* expression and chlorophyll accumulation has not been elucidated. In this study, we observed that shading adversely affects CsHY5 to prevent it from inhibiting the expression of the downstream *CsPOR* genes, thereby promoting the accumulation of chlorophyll (Figures 4A, 6C). A previous study on *A. thaliana* indicated that HY5 may negatively regulate the expression of *POR* genes (Lee et al., 2007). This earlier result combined with the data generated in this study indicate that *POR* expression may be regulated by HY5 in multiple species. In addition to HY5, PIFs are important transcription factors in the light signaling pathway and they also regulate the expression of chlorophyll biosynthesis genes (Moon et al., 2008). In *A. thaliana*, both PIF1 and PIF3 help regulate *POR* expression. Additionally, PIF1 can bind directly to the *PORC* promoter to modulate expression (Cheminant et al., 2011). In the current study, we determined that CsPIF3-2 has no regulatory effects on *POR* expression, which is in contrast to the inhibited *POR* transcription mediated by PIF7-1 (Figure 6C). Accordingly, the transcriptional regulation of *POR* genes by PIFs appears to be conserved in multiple species, but the specific regulators may differ among species. Interestingly, CsPIF7-1 negatively regulated the expression of *CsPORL-2*, which seemed were not consistent with the results of accumulation of chlorophyll and the expression of *CsPIF7-1* under shading treatment. However, recent study showed that shade-tolerant *Cardamine hirsuta* had higher HFR1 activity, which inhibited more effectively PIF action than *Arabidopsis thaliana* (Paulišić et al., 2021). As a result, a reduced PIF activity and attenuating other PIF-mediated responses were observed under shading. Since tea is also shade-tolerant plant, there may be other regulators, such as HFR1, that inhibit the activity of PIF and the regulation of downstream genes by PIF under shading. Therefore, further work needs to find upstream regulators of PIF7-1. In addition, PIF abundance is attenuated in *C. hirsute* (Paulišić et al., 2021). CsPIF7-1 as a transcription factor to regulate downstream gene expression depends on its protein stability, the protein enrichment of CsPIF7-1 under long time shading treatment was still unclear and needed further investigation. In addition, there may indeed be other regulatory mechanisms between *CsPIF7-1* and *CsPORL-2* that we do not yet know. Collectively, the regulatory roles of these transcription factors will need to be more precisely characterized.

Previous research confirmed that shading decreases the light intensity and the ratio of R: FR, which will affect plant growth and development (Fiorucci and Fankhauser, 2017). All higher plants perceive R: FR changes through members of the phytochrome photoreceptor family; this is often regarded as an indicator of the presence of nearby competitors. On the basis of their responses to shading, plants can be categorized as shade-sensitive plants that exhibit shade-avoidance responses and shade-tolerant plants (Fiorucci and Fankhauser, 2017). Shade-avoidance responses include the elongation of stems or petioles rather than branching, the increased vertical positioning of leaves (i.e., to the top of the canopy), and earlier blooming. These changes are collectively known as the shade-avoidance syndrome. In contrast, shade-avoidance responses are absent in shade-tolerant plants, which thrive on the forest floor and do not grow taller than the surrounding trees (Gommers et al., 2017). *A. thaliana* is considered to be a shade-sensitive plant species, whereas tea plants are generally considered to be shade-tolerant species. Furthermore, previous research proved that shading increases the chlorophyll a and b contents of tea plants, but the ratio of chlorophyll a: chlorophyll b was decreased and the expression levels of the corresponding photosynthesis-related genes were upregulated (Liu et al., 2020). Similar results were obtained in this study (Figure 1C). These changes reflect the typical shade tolerance response. However, compared with the extensive research on the shade-avoidance responses of plants, there have been relatively few investigations regarding the mechanism underlying plant shade tolerance. Studies involving several shade-tolerant species demonstrated that specialized epidermal chloroplasts, altered gene expression patterns, and the increased efficiency of phyA-dependent pathways may enable shade-tolerant plants to adapt to low R: FR light environmental conditions (Cheminant et al., 2011; Jacobs et al., 2016; Gommers et al., 2017). Future analyses of the shade tolerance of tea plants will clarify the effects of shading on tea plants, while also elucidating the adaptive mechanism underlying the shade tolerance of plants.

**Figure 7:**
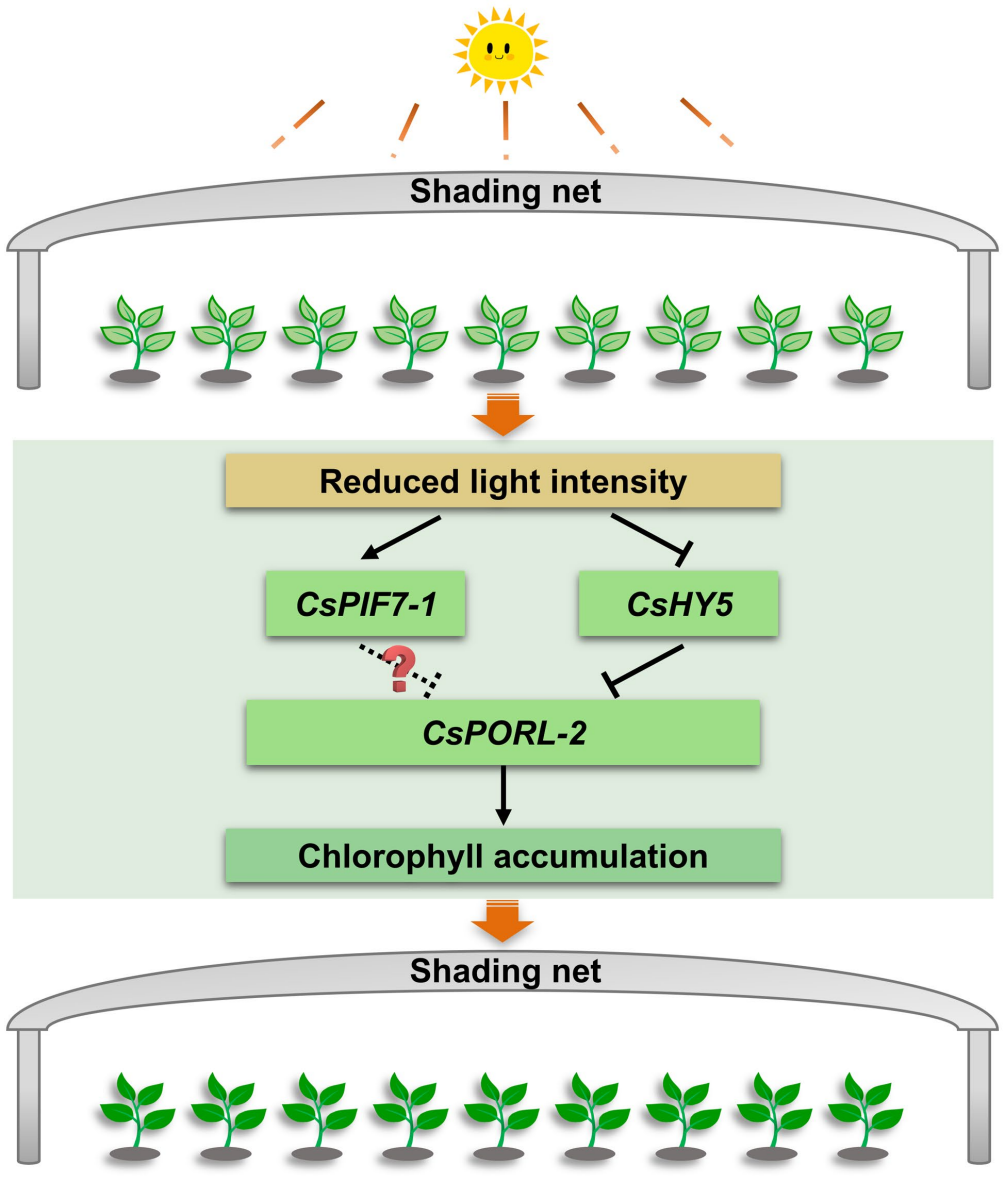
The regulation mechanism model of chlorophyll accumulation under shade treatment of tea plants. After shading, the decrease of light intensity affects the transcription level of *CsHY5* and *CsPIF7-1*, which in turn regulates the expression of the chlorophyll synthesis gene *CsPORL-2*, and causes the accumulation of chlorophyll in tea leaves. Arrows and bars indicate positive regulation and negative regulation, respectively. The solid line indicates the regulatory mechanism in this study, while dashed line and question mark indicate unknown mechanisms.

This study explored the molecular basis of the shade-induced regulation of chlorophyll accumulation in tea leaves. The results were used to develop the following model (Figure 7). Shading significantly decreases the light intensity, which is the key environmental factor that promotes the accumulation of chlorophyll in tea leaves. Moreover, the decreased light intensity significantly downregulates and upregulates the expression of *CsHY5* and *CsPIF7-1*, respectively. The decreased abundance of CsHY5 leads to the enhanced expression of *CsPORL-2*, which encodes a key rate-limiting enzyme involved in chlorophyll synthesis, ultimately resulting in chlorophyll accumulation. While CsPIF7-1 may also be involved in regulating the expression of *CsPORL-2* through an unknown mechanism, thereby affecting the formation of chlorophyll, but the specific regulatory mechanism needs to be further studied. These results advance our understanding of the molecular regulatory mechanism of chlorophyll accumulation in tea plant under shade treatment and provide reference for the analysis of stress tolerance of tea plant. In addition, this information will contribute to the breeding of tea varieties with high chlorophyll.

## Data Availability Statement

The datasets presented in this study can be found in online repositories. The names of the repository/repositories and accession number(s) can be found in the article/Supplementary Material.

## Author Contributions

DG conceived and designed the experiments. JC, SW, JL, and LZ conducted the experiments. FD, JL, LZ, JT, and DG analyzed the results. JC, SW, and DG wrote the manuscript. All authors reviewed the manuscript.

## Funding

This study was supported by the Guangdong Basic and Applied Basic Research Foundation (2020A1515010539), the Medical Science and Technology Research Foundation of Guangdong Province, China (A2019046), the Basic Frontier Science Research Program of Chinese Academy of Sciences (ZDBS-LY-SM032), and the Guangdong Provincial Special Fund for Modern Agriculture Industry Technology Innovation Teams (2020KJ120).

## Conflict of Interest

The authors declare that the research was conducted in the absence of any commercial or financial relationships that could be construed as a potential conflict of interest.

## Publisher’s Note

All claims expressed in this article are solely those of the authors and do not necessarily represent those of their affiliated organizations, or those of the publisher, the editors and the reviewers. Any product that may be evaluated in this article, or claim that may be made by its manufacturer, is not guaranteed or endorsed by the publisher.

## Footnotes

^1^ https://bioinfo.ut.ee/primer3-0.4.0/

^2^ https://blast.ncbi.nlm.nih.gov/

## Supplementary Material

Supplementary Figure S1 Effect of shading treatment on the chlorophyll content of tea leaves and the expression of related genes. **(A)** The phenotype of new shoots after shading treatment. Bar=2 cm. **(B)** The change of chlorophyll content after shading treatment. Chl: total chlorophyll; Chl a: chlorophyll a; Chl b: chlorophyll b; and FW: fresh weight. **(C)** The changes in the gene expression of *CsPORL-2* after shading treatment. **(D)** Changes in the expression of genes related to light signal in tea plants under shading treatment. CK: no shading treatment group (about 1,300 μmol·m^−2^·s^−1^); T: 90% shading treatment group (about 130 μmol·m^−2^·s^−1^). *CsEF1-α* was used as an internal reference to normalized the changes. Data are expressed as mean±SD (*n*=3). **p*≤0.05; ***p*≤0.01; and difference from CK treatment at the same time point.
