# Supplementary figures and images for: Mechanism Underlying the Shading-Induced Chlorophyll Accumulation in Tea Leaves
Source: Front Plant Sci. 2021 Dec 2;12:779819. doi: 10.3389/fpls.2021.779819 (PMC8675639; doi:10.3389/fpls.2021.779819)

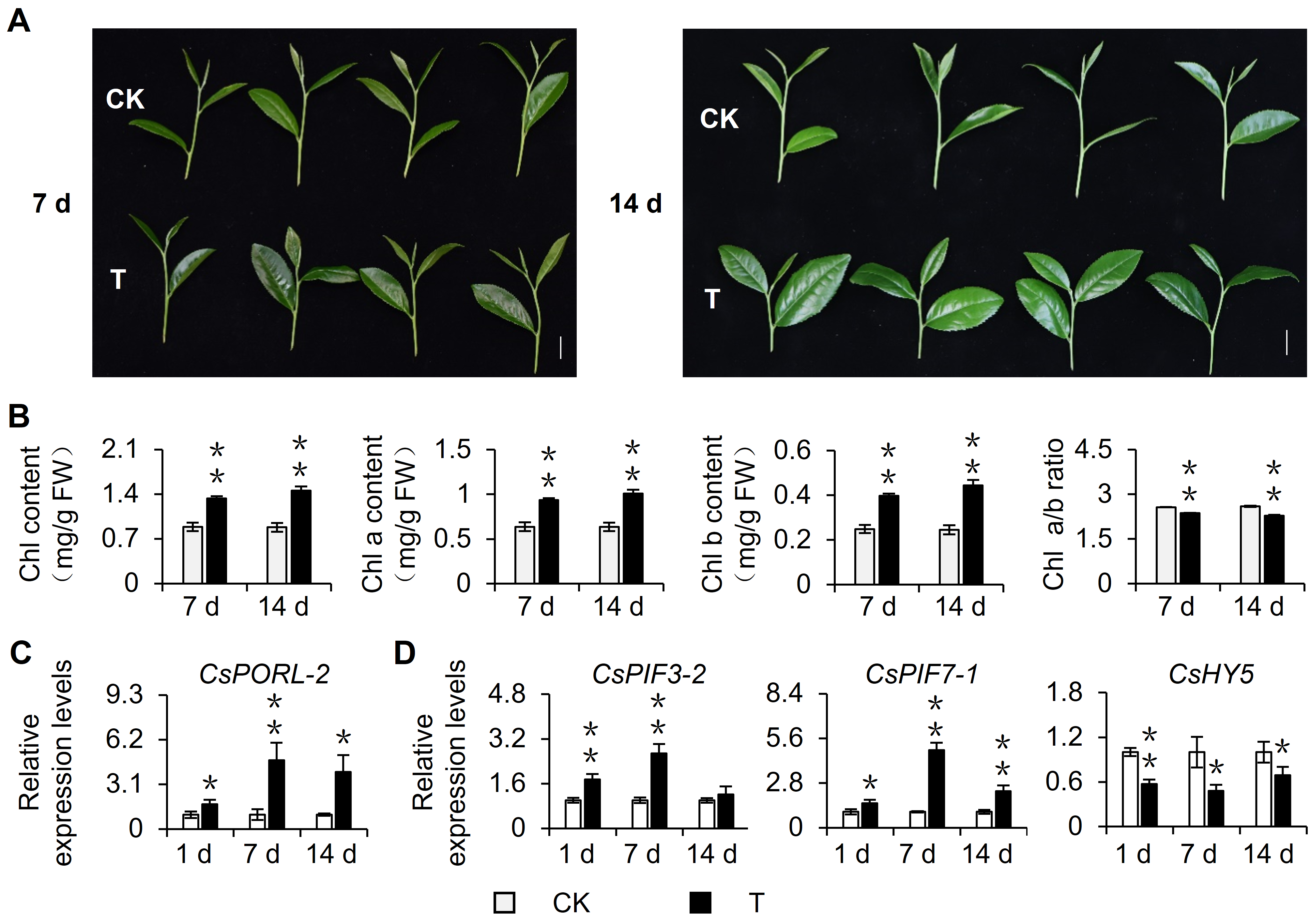

Supplement: Supplementary Figure S1 — Effect of shading treatment on the chlorophyll content of tea leaves and the expression of related genes. (A) The phenotype of new shoots after shading treatment. Bar=2 cm. (B) The change of chlorophyll content after shading treatment. Chl: total chlorophyll; Chl a: chlorophyll a; Chl b: chlorophyll b; and FW: fresh weight. (C) The changes in the gene expression of CsPORL-2 after shading treatment. (D) Changes in the expression of genes related to light signal in tea plants under shading treatment. CK: no shading treatment group (about 1,300 μmol·m−2·s−1); T: 90% shading treatment group (about 130 μmol·m−2·s−1). CsEF1-α was used as an internal reference to normalized the changes. Data are expressed as mean±SD (n=3). *p≤0.05; **p≤0.01; and difference from CK treatment at the same time point. [file Image_1.TIF]
