# Supplementary material for: Mechanism Underlying the Shading-Induced Chlorophyll Accumulation in Tea Leaves
Source: Front Plant Sci. 2021 Dec 2;12:779819. doi: 10.3389/fpls.2021.779819 (PMC8675639; doi:10.3389/fpls.2021.779819)
Supplement: Supplementary file 2 [file Table_1.DOCX]

Supplementary Material

**Table S1.** The primers pairs used in this study

| Gene name | Accession number | Forward primer 5'-3' | Reverse primer 5'-3' |
| --- | --- | --- | --- |
| Primer pairs for qRT-PCR |  |  |  |
| *CsEF1-α* | KA280301.1 | TTCCAAGGATGGGCAGAC | TGGGACGAAGGGGATTTT |
| *CsPIF1* | XM_028209377.1 | GAGCCAAAACCAGAGATCCA | TAGTATGGGCGAGAGGATCG |
| *CsPIF3-1* | XM_028252927.1 | GAAGTGATGGCGGGTAAGAA | CAGCTGCCACTGATTTTGAA |
| *CsPIF3-2* | XM_028207807.1 | ATGGCTAAGCGACTTGAGGA | CTATTGCCAGAACACGCAGA |
| *CsPIF7-1* | XM_028228667.1 | CGCTGGAATCCATAGTCCAT | ATTCTGACCACATCCGCTTC |
| *CsPIF7-2* | XM_028254717.1 | AAACAGATGCGGTCGAAATC | TCTCGCTCTTCATCCAGGTT |
| *CsPIF8-1* | XM_028267851.1 | GCTGCCTTCATGCCTCTAAC | CAAGGCTGCCATCCTGTTAT |
| *CsPIF8-2* | XM_028264035.1 | TGACCTTGCAGCAACAACTC | CAAGAAGGTAGGGGCATGAA |
| *CsHY5* | XM_028265046.1 | CCAGCTCTGCTCTTCAGCTT | CTTGTCAGCCGGACTTCTTC |
| *CsHEMA1* | XM_028228202.1 | TGCAGCTGACAGGTATACAAA | ATGGCAAGCTTTTCACGCATT |
| *CsHEMA2* | KAF5955413.1 | GGAAGTTGGGGGTCTTAGGC | CGTTATAAACCCGTGTGGCG |
| *CsCHLH* | XM_028213021.1 | CCCAATTCAGGTGGCTTTGC | TGAAGAGCATGTGATTTCCCTGT |
| *CsCHLI1* | XM_028225249.1 | GTGTTCTCGGAACTTCTTCTGC | CATAACTCTGCCCTGGGGTT |
| *CsCHLD* | XM_028232541.1 | CGTTGTCGGACAGGATGCTA | CTGTTTTGGCTGTTCCTCGC |
| *CsDVR1* | XM_028251252.1 | TGCTAGGGAGAGTAGTGGCA | TGGGTCACATCCGAAAAGCA |
| *CsDVR2* | XM_028258564.1 | AAGCCGATTAGTGAGCCTGA | ACCAATGGGCAAAACCCTGT |
| *CsPOR* | XM_028215906.1 | GGCTCCATAACAGGAAACACA | TTCAAGCCTCCTGCAAGTCC |
| *CsPORL-1* | XM_028228181.1 | AGGGAAAGCCTAGTGTTTCTTTGA | GCCATTGGTTTGATTCCTTCTTGA |
| *CsPORL-2* | XM_028200153.1 | CAGTCTGCTGCTGCTGCTAC | TGGATACGTTGATTGCTGGA |
| *CsCAO* | XM_028237062.1 | GCAACAAACGGGGTGTTAGG | TCAAAGAGAGTGCCCCATGC |
| *CsCHLG* | XM_028265445.1 | CATAGCTGGGCTGGGCATT | GCAGTCTCCGAACCAAAAGC |
| Primer pairs used for constructions |  |  |  |
| *CsPIF3-2- pHB-FLAG infusion* | XM_028207807.1 | CTCTCTCTCAAGCTTGGATGCCTTTCTCAGAG | CCGTCACTAGTGGATCCGCTACTGGCATCAGC |
| *CsPIF7-1- pHB-FLAG infusion* | XM_028228667.1 | CTCTCTCTCAAGCTTGGATGAAAGGAATCATG | CCGTCACTAGTGGATCCACCCCCTTGAACATG |
| *CsHY5- pHB-FLAG infusion* | XM_028265046.1 | CTCTCTCTCAAGCTTGGATGCAAGAACAAGCA | CCGTCACTAGTGGATCCCTTCCTACCCTCCTG |
| *CsPIF3-2- pSAT6-EYFP infusion* | XM_028207807.1 | CGGTACCGCGGGCCCGGGATGCCTTTCTCAGAG | CACCATCAGGATCCCGGGGCTACTGGCATCAGC |
| *CsPIF7-1- pSAT6-EYFP infusion* | XM_028228667.1 | CGGTACCGCGGGCCCGGGATGAAAGGAATCATG | CACCATCAGGATCCCGGGACCCCCTTGAACATG |
| *CsPIF7-2- pSAT6-EYFP infusion* | XM_028254717.1 | CGGTACCGCGGGCCCGGGATGAGTGAGTGGATA | CACCATCAGGATCCCGGGTCCACCTTGGACATG |
| *CsHY5-pSAT6-EYFP infusion* | XM_028265046.1 | CGGTACCGCGGGCCCGGGATGCAAGAACAAGCA | CACCATCAGGATCCCGGGCTTCCTACCCTCCTG |
| *CsPORL-2pro-reporter* | XM_028200153.1 | GACGGTATCGATAAGCTTTATTTTTGTGTAGGGAA | TCTAGAACTAGTGGATCCAGACTGGAGAGCCAT |

*EF1-α*, *encoding elongation factor 1-α*; *PIFs*, *phytochrome interaction factors*; *HY5*, *elongated hypocotyl 5*; *HEMA*, *encoding glutamyl-tRNA reductase; CHLH, encoding Magnesium chelatase H subunit; CHLI, encoding Magnesium chelatase Ⅰ subunit; CHLD, encoding Magnesium chelatase D subunit; DVR, encoding 3,8-divinyl Chlide 8-vinyl reductase; POR, encoding protochlorophyllide oxidoreductase; CAO,* *encoding Chlorophyllide a oxygenase; CHLG, encoding* *Chlorophyll synthase.*
